# An Immuno-Fragile Profile Is Associated with Mortality Risk in Patients with Chronic Kidney Disease

**DOI:** 10.3390/biomedicines13102370

**Published:** 2025-09-27

**Authors:** Noemí Ceprián, Irene Martínez de Toda, Paula Jara Caro, Claudia Yuste, Gemma Valera-Arévalo, Ignacio González de Pablos, Andrea Figuer, Matilde Alique, Rafael Ramírez, Enrique Morales, Julia Carracedo

**Affiliations:** 1 Departamento de Genética, Fisiología y Microbiología, Facultad de Ciencias Biológicas, Universidad Complutense de Madrid, 28040 Madrid, Spain; gvalera@ucm.es (G.V.-A.); julcar01@ucm.es (J.C.); 2 Instituto de Investigación Sanitaria Hospital 12 de Octubre (imas12), RICORS 2040, 28041 Madrid, Spain; jcaroespada@gmail.com (P.J.C.); claudiayustelozano@yahoo.es (C.Y.); emoralesr@senefro.org (E.M.); 3 Departamento de Nefrología, Hospital Universitario 12 de Octubre, RICORS 2040, 28041 Madrid, Spain; igp.snurse@gmail.com; 4 Departamento de Biología de Sistemas, Universidad de Alcalá, 28871 Alcalá de Henares, Spain; andrea.figuer@salud.madrid.org (A.F.); matilde.alique@uah.es (M.A.); manuel.ramirez@uah.es (R.R.); 5 Instituto Ramón y Cajal de Investigación Sanitaria (IRYCIS), 28034 Madrid, Spain; 6 Departamento de Medicina, Facultad de Medicina, Universidad Complutense de Madrid, 28040 Madrid, Spain

**Keywords:** immune alterations, frailty, immune-fragile profile, mortality risk, advanced chronic kidney disease, hemodialysis, peritoneal dialysis, kidney transplantation

## Abstract

**Background/Objectives:** Patients with chronic kidney disease (CKD) face higher risks of infections, poor vaccine responses, and cardiovascular diseases, leading to increased morbidity and mortality due to immune dysfunction and frailty. This study aims to evaluate immune status and frailty in CKD patients across different treatments, examine the influence of frailty on immune status, and link these factors to mortality. **Methods**: A total of 174 participants were included (end-stage renal disease, ESRD n = 40; hemodialysis, HD n = 40; peritoneal dialysis, n = 36; kidney transplant patients, n = 40; healthy subjects n = 18). Immunophenotyping of lymphocyte and monocyte subpopulations was performed, and frailty was assessed using the Edmonton Frail Scale. Principal component analysis (PCA) integrated immune and frailty variables to define an “immuno-fragile profile,” and survival was monitored for up to six years. **Results**: CKD patients, especially those on HD, showed decreased lymphocyte counts and proinflammatory monocyte subpopulations with increased expression of costimulatory molecules (B7.2/CD86 and ICAM-1/CD54). Frailty was most prevalent in HD patients (53%), with notable sex differences. PCA identified three components—lymphocyte counts, monocyte co-stimulatory expression, and frailty—that together explained 70% of the variance. Survival analysis revealed that patients with lower lymphocyte counts and higher frailty scores had increased mortality risk, especially in the HD and ESRD groups. Cox regression confirmed that the immuno-fragile profile independently predicted mortality. **Conclusions**: The integration of immune alterations and frailty defines an immuno-fragile profile strongly associated with mortality in CKD patients, which may serve as a robust prognostic tool to improve risk stratification and guide personalized interventions in clinical practice.

## 1. Introduction

Chronic kidney disease (CKD) is a significant public health issue and one of the leading causes of death, affecting over 10% of the global population [1,2,3]. Recently, the incidence and prevalence of CKD have risen due to an increase in risk factors such as diabetes mellitus, hypertension, obesity, and aging [4,5,6,7,8].

CKD is considered a disease that occurs with accelerated and premature aging [9,10,11]. Patients with CKD display traits similar to those observed with aging, such as a high rate of cardiovascular disorders, the establishment of low-grade chronic inflammatory stress, immunosenescence, oxidative stress, osteoporosis, and increased frailty, all of which contribute to a shorter life expectancy compared to the general population [7,9,11,12,13].

In this context, the leading causes of death in these patients are cardiovascular disease and increased susceptibility to infections, both of which are closely related to the immune system [8,14,15,16,17]. The fibrosis process in the kidney, along with rising levels of uremic toxins in the blood (notably indoxyl sulfate and p-cresol), leads to immunosenescence, chronic low-grade inflammation or inflammaging, increased oxidative stress, endothelial dysfunction, and disruption of the gut barrier with microbial translocation [14,18,19,20,21]. In turn, this results in lymphopenia, reduced naïve T and B cell populations, an imbalance in the Th1/Th2 ratio, elevated proinflammatory cytokine production, and impaired antigen-specific responses [4,15,18,20,22,23,24,25]. These factors contribute to a higher frequency and severity of infections, decreased vaccine effectiveness, and impaired tumor surveillance [13,24,25,26,27,28].

Furthermore, immunosenescence, inflammaging, and oxidative stress in CKD patients cause endothelial dysfunction and senescence, as well as the expansion of proinflammatory monocyte subsets (intermediate CD14++CD16+ and non-classical CD14+CD16+ monocytes), which exhibit impaired regulation of apoptosis [14,29,30]. Additionally, these monocytes express high levels of adhesion molecules, which promote vascular infiltration and contribute to microinflammation, plaque formation, and endothelial senescence [31,32,33]. All of these factors increase the risk of atherosclerosis and cardiovascular disease in these patients.

CKD patients are especially vulnerable to frailty [34]. Frailty is an age-related condition characterized by decreased homeostatic capacity resulting from declining physiological function and increasing susceptibility to stress, illness, and death [34,35]. Frail individuals may face hospitalization or disability from minor stresses [36]. It is a major predictor of adverse outcomes such as falls, fractures, and reduced quality of life, leading to increased healthcare utilization and polypharmacy [36,37,38]. However, the underlying mechanisms of frailty are still not well understood. Contributing factors may include chronic inflammation, immune activation, anemia, low hematocrit levels, obesity, and other comorbidities [38]. In CKD, research shows that the risk of frailty increases as glomerular filtration rate decreases [39], especially among elderly patients, women, and those with diabetes [40]. Indeed, frailty is an independent risk factor for death or progression to dialysis [34].

Given the significance of immune disorders and frailty on the health and quality of life of CKD patients, this study aimed to assess the immune status in patients with end-stage renal disease, undergoing hemodialysis, peritoneal dialysis, or kidney transplantation, compared to healthy subjects. The relationship between immune status and frailty was also examined across different patient groups.

## 2. Materials and Methods

### 2.1. Study Design, Patient Population

This observational study included both cross-sectional and prospective components. It involved a total of 116 CKD participants from three distinct treatment groups: end-stage renal disease (n = 40, ESRD) stage 4–5, hemodialysis (n = 40, HD), and peritoneal dialysis (n = 36, PD). Additionally, 40 participants were undergoing initial kidney transplantation (KT), and 18 healthy subjects (HS) were included. All participants were stable and had been receiving the same treatment for at least six months. Those with neoplasms, infections, or active inflammatory or autoimmune diseases were excluded. The research was conducted at the Nephrology Department of Hospital Universitario “12 de Octubre” in Madrid, Spain, from May 2018 to March 2022. Participants who wished to continue in the study were monitored until September 2024, with the date and cause of death recorded if applicable. At the end of the study, 28 participants were lost due to their decision to leave or change medical centers, including 10 participants with ESRD, 10 with HD, 6 with PD, and 2 with KT. A summary of the experimental design and patients at each stage is presented in Figure S1. All procedures were conducted in accordance with the Declaration of Helsinki, as adopted by the World Medical Association, and the Declaration of Istanbul, endorsed by the Transplantation Society and the International Society of Nephrology. The protocol received approval from the Ethics Committee of Instituto de Investigación Sanitaria Hospital 12 de Octubre (CEIC: 17/407). All patients provided written informed consent.

### 2.2. Sample Collection and Measurements

Peripheral blood samples were collected from patients and healthy subjects using ethylenediaminetetraacetic acid (EDTA)-coated tubes. Samples were collected during periodic medical reviews and, in the case of hemodialysis patients, prior to initiating hemodialysis. All samples were analyzed within 18 h.

Biochemical and lymphocyte population analyses were performed at the Department of Clinical Analysis and the Department of Immunology at “12 de Octubre” Hospital. Monocyte characterization was conducted at the Department of Genetics, Physiology, and Microbiology at the Complutense University of Madrid, Spain. Lymphocyte and monocyte phenotyping followed established protocols [41]. Total lymphocytes, T cells (CD3+), T-helper cells (CD3+CD4+), T-cytotoxic cells (CD3+CD8+), B cells (CD19+), and NK cells (CD56+) were quantified using a FACSCanto II cytometer (BD Biosciences, San Jose, CA, USA) and FACSDiva software v8 (BD Biosciences). For monocytes, the percentages of classical (CD14++CD16-), intermediate (CD14++CD16+), and non-classical (CD14+CD16+) subtypes were evaluated, along with the expression of co-stimulatory molecules ICAM-1 (CD54+) and B7.2 (CD86+). They were analyzed at the Flow Cytometry and Fluorescence Microscopy Core Facility of the Complutense University of Madrid, using a FACSCalibur cytometer (BD Biosciences) and FlowJosoftware v10 (Tree Star, Ashland, OR, USA).

Patient frailty was evaluated using the Edmonton Frail Scale (EFS) [42], which has been validated in the Spanish language and assesses nine domains: cognition, health status, functional independence, social support, medication use, nutrition, mood, continence, and performance. Patients complete an eleven-question questionnaire, and their frailty level is determined by a score out of 17, with higher scores indicating greater frailty. According to the official EFS website (Edmontonfrailscale.org), categories include Fit (0–3), Vulnerable (4–5), Mildly Frail (6–7), Moderately Frail (8–9), and Severely Frail (≥10).

### 2.3. Statistical Analysis

Statistical analysis was conducted using SPSS v28.0 (IBM Corp., Armonk, NY, USA). Categorical variables were compared with the chi-squared test. In contrast, continuous variables were analyzed using the Kruskal–Wallis test, with adjusted post hoc pairwise comparisons, and presented as mean ± SD or range. Principal component analysis (PCA) was applied to selected immune parameters and frailty scores, retaining components with eigenvalues greater than 1 and employing varimax rotation. PCA variables were selected beforehand based on clinical or biological relevance in CKD, immunosenescence, and frailty (lymphocyte subsets, monocyte activation markers, and frailty score), as well as empirical contribution in preliminary analyses (retaining indicators that provided non-redundant information). Survival analysis was performed with Kaplan–Meier curves and the log-rank test. A multivariable Cox proportional hazards model was used to examine the relationship between PCA-derived components and mortality, adjusting for conventional prognostic factors (age, sex, diabetes, and baseline cardiovascular disease). Hazard ratios (HR) and *p*-values were reported, with *p* ≤ 0.05 considered statistically significant.

## 3. Results

### 3.1. Characteristics of Participants

Table 1 summarizes the clinical and demographic characteristics of the patients. Although age, sex, and etiopathology were similar across the groups, patients with end-stage renal disease (ESRD) had a higher average age (61 ± 17 years) than healthy subjects (HS, 51 ± 16 years, *p* = 0.02). Body mass index (BMI) was higher in ESRD (27.4 ± 5.4) and kidney transplant (KT) patients (27.0 ± 5.2) compared to HS (24.6 ± 3.6, *p* = 0.04 vs. ESRD), hemodialysis (HD) (24.3 ± 4.0, *p* = 0.01 vs. ESRD, *p* = 0.02 vs. KT), and peritoneal dialysis (PD) (24.8 ± 4.1, *p* = 0.03 vs. ESRD).

Cardiovascular risk factors, including hypertension and dyslipidemia, were consistent across groups: ESRD (90% and 78%), HD (83% and 58%), PD (92% and 61%), and KT (98% and 53%). Notably, diabetes mellitus was more common in ESRD (45%, *p* = 0.01) and KT (40%, *p* = 0.03) than in HD (18%).

The biochemical analysis (Table 2) revealed lower serum albumin (ESRD: 4.3 ± 0.4; HD: 4.1 ± 0.4; PD: 3.8 ± 0.5), total proteins (HD: 6.7 ± 0.7; PD: 6.4 ± 1.4), and hemoglobin (ESRD: 11.7 ± 2.2; HD: 12 ± 1.6; PD: 11.7 ± 1.5) levels in chronic kidney disease (CKD) patients compared to HS and KT recipients (*p* < 0.001). Elevated serum creatinine levels were observed in all CKD patients (ESRD: 4.3 ± 1.2; HD: 7.8 ± 1.9; PD: 7.3 ± 2.5) compared to HS (0.8 ± 0.2, *p* < 0.001 vs. ESRD, HD, PD; *p* = 0.04 vs. KT). Uric acid levels were higher in ESRD (6.5 ± 1.9, *p* = 0.01) and KT (6.9 ± 1.5, *p* < 0.001) than in HS (5.0 ± 1.2). C-reactive protein levels were also increased in all patient groups (ESRD: 0.5 ± 0.4, *p* = 0.01; HD: 0.9 ± 2.1, *p* < 0.001; PD: 0.9 ± 1.9, *p* = 0.01; KT: 0.5 ± 0.9, *p* = 0.03) compared to controls (0.3 ± 0.5).

### 3.2. Immune Cells Characterization

**Figure 1:**
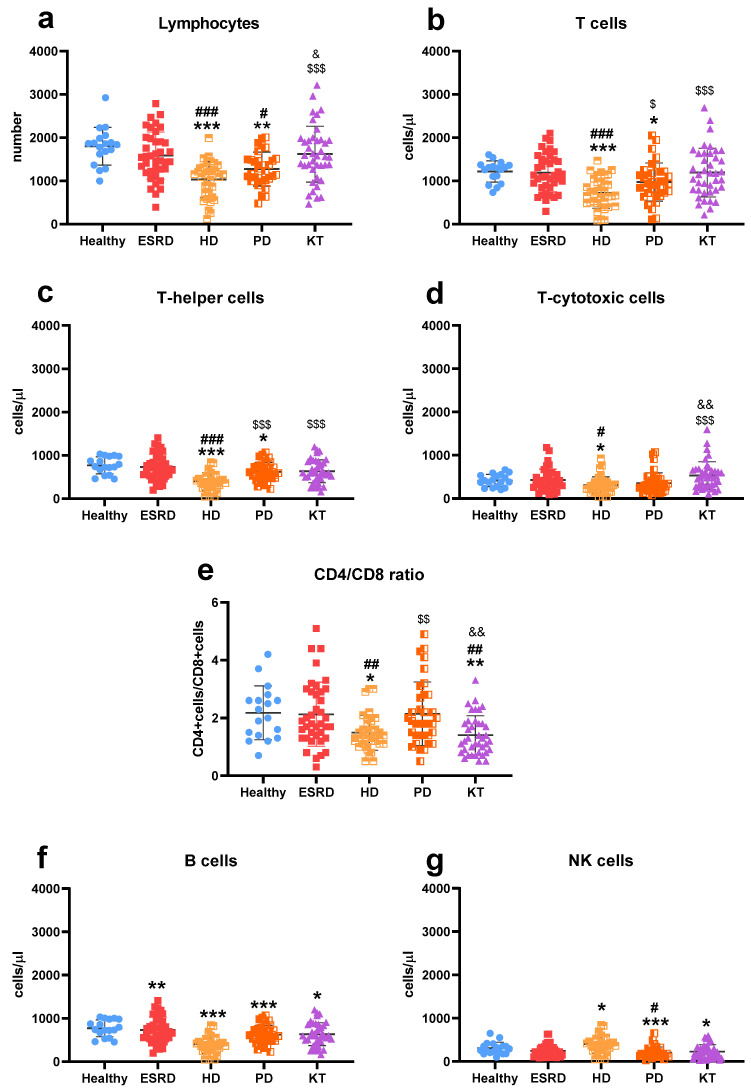
Description of lymphocyte subpopulations. Number of total lymphocytes (**a**), t-cells (**b**), t-helper cells (CD4) (**c**), and t-cytotoxic cells (CD8) (**d**), the relation between helper and cytotoxic cells (CD4/CD8 ratio) (**e**), number of B cells (**f**) and natural killer (NK) cells (**g**) in healthy subjects, patients with end-stage renal disease (ESRD), hemodialysis (HD), peritoneal dialysis (PD), and kidney transplantation (KT). Statistical significance was denoted by * *p* ≤ 0.05, ** *p* ≤ 0.01, *** *p* ≤ 0.001 vs. HS; ^#^ *p* ≤ 0.05, ^##^ *p* ≤ 0.01, ^###^ *p* ≤ 0.001 vs. ESRD; ^$^ *p* ≤ 0.05, ^$$^ *p* ≤ 0.01, ^$$$^ *p* ≤ 0.001 vs. HD; ^&^ *p* ≤ 0.05, ^&&^ *p* ≤ 0.01 vs. PD.

The number of lymphocytes (Figure 1a) in the blood was lower in dialysis patients (HD: 1033 ± 410, PD: 1274 ± 392) compared to HS (1737 ± 345, *p* < 0.001 and *p* = 0.01, respectively), ESRD (1585 ± 549, *p* < 0.001 and *p* = 0.02, respectively), and KT (1620 ± 647, *p* < 0.001 and *p* = 0.01, respectively). T cell levels (Figure 1b; HS: 1218 ± 247, ESRD: 1188 ± 440, HD: 721 ± 351, PD: 975 ± 440, KT: 1195 ± 563), especially T-helper cells (Figure 1c; HS: 770 ± 194, ESRD: 732 ± 296, HD: 398 ± 206, PD: 625 ± 214, KT: 1195 ± 563), were lower in PD than in HS (*p* = 0.03 and *p* = 0.05), and in HD compared to other groups (*p* < 0.001 in all cases minus T-cell levels vs. PD with *p* = 0.02). HD patients (308 ± 191) had fewer T-cytotoxic cells (Figure 1d) than healthy subjects (417 ± 142, *p* = 0.02), ESRD (429 ± 251, *p* = 0.02), and KT patients (531 ± 321, *p* < 0.001). All these findings resulted in a lower helper/cytotoxic ratio (Figure 1e) in HD (1.5 ± 0.6) and KT (1.5 ± 1.0) compared to HS (2.2 ± 0.9, *p* = 0.011 and *p* = 0.01), ESRD (2.1 ± 1.1, *p* = 0.01 and *p* = 0.002), and PD (2.1 ± 1.1, *p* = 0.01 and *p* = 0.002). The number of B cells (Figure 1f) was decreased in all patients (ESRD: 131 ± 116, *p* = 0.002; HD: 119 ± 125, *p* < 0.001; PD: 95 ± 64, *p* < 0.001; KT: 137 ± 93, *p* = 0.02) compared to healthy subjects (199 ± 87). Similarly, NK cell counts (Figure 1g; HS: 303 ± 140; HD: 203 ± 129, *p* = 0.01; PD: 160 ± 80, *p* < 0.001; KT: 225 ± 161, *p* = 0.02) were also reduced.

**Figure 2:**
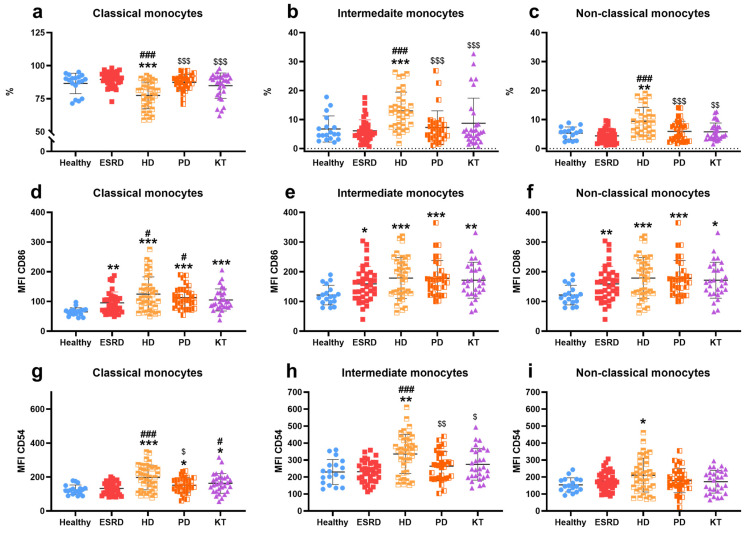
Description of monocyte subpopulations. Percentage of classical (**a**), intermediate (**b**), and non-classical (**c**) monocytes; the amount of B7.1/CD86 expressed by classical (**d**), intermediate (**e**), and non-classical (**f**) monocytes, and the amount of ICAM-1/CD54 expressed by classical (**g**), intermediate (**h**), and non-classical (**i**) monocytes in healthy subjects, patients with end-stage renal disease (ESRD), hemodialysis (HD), peritoneal dialysis (PD), and kidney transplantation (KT). MFI: mean fluorescence intensity. Statistical significance was denoted by * *p* ≤ 0.05, ** *p* ≤ 0.01, *** *p* ≤ 0.001 vs. HS; ^#^ *p* ≤ 0.05, ^###^ *p* ≤ 0.001 vs. ESRD; ^$^ *p* ≤ 0.05, ^$$^ *p* ≤ 0.01, ^$$$^ *p* ≤ 0.001 vs. HD.

The percentage of classical monocytes (Figure 2a) was reduced in HD (77 ± 10) compared to other groups (HS: 87 ± 8, ESRD: 90 ± 5, PD: 87 ± 6, KT: 85 ± 10, *p* ≤ 0.001 in all cases). Meanwhile, the percentages of intermediates (Figure 2b) and non-classical monocytes (Figure 2c) increased in HD (13 ± 6 and 9 ± 5, respectively) (HS: 7 ± 5, *p* < 0.001; 5 ± 2, *p* = 0.01; ESRD: 6 ± 4, *p* < 0.001; 4 ± 2, *p* < 0.001; PD: 7 ± 6, *p* < 0.001; 6 ± 4, *p* < 0.001; KT: 9 ± 9, *p* < 0.001; 6 ± 3, *p* = 0.003). Additionally, the expression levels of CD86/B7.2 on classical (Figure 2d), intermediate (Figure 2e), and non-classical (Figure 2f) monocytes increased in ESRD (95 ± 36, *p* = 0.002; 159 ± 59, *p* = 0.01; 157 ± 51, *p* = 0.01), HD (125 ± 58, *p* < 0.001; 179 ± 69, *p* < 0.001; 181 ± 80, *p* < 0.001), PD (113 ± 35, *p* < 0.001; 178 ± 60, *p* < 0.001; 188 ± 58, *p* < 0.001), and KT (105 ± 37, *p* < 0.001; 172 ± 61, *p* = 0.002; 161 ± 79, *p* = 0.01) compared to HS (65 ± 13, 122 ± 33, 119 ± 25). Regarding ICAM-1/CD54 expression by monocytes, it was higher in hemodialysis patients (197 ± 73, *p* < 0.001; 335 ± 114, *p* < 0.001; 210 ± 99, *p* = 0.02) across all three monocyte subtypes (Figure 2g–i) versus HS (125 ± 28, 229 ± 74, 153 ± 42). Furthermore, the expression of this molecule was increased in PD (152 ± 45, *p* = 0.04) and KT (163 ± 59, *p* = 0.02) in classical monocytes compared to HS. Lastly, the percentage of B7.2/CD86 positive monocytes was higher in patients (Figure S2a–c), and the rate of ICAM-1/CD54 positive monocytes was elevated in HD and KT compared to HS (Figure S2d–f) in classical and intermediate monocyte populations.

### 3.3. Frailty

**Figure 3:**
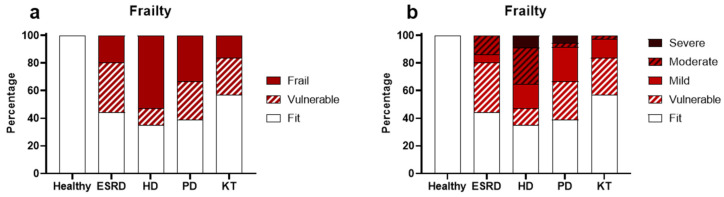
Frailty status of patients. The frailty status in general (**a**) or subdivided by the frail degree (**b**) of healthy subjects, patients with end-stage renal disease (ESRD), hemodialysis (HD), peritoneal dialysis (PD), and kidney transplantation (KT).

The patients’ frailty status, as assessed by the Edmonton Frail Scale (EFS), is shown in Figure 3. A higher proportion of patients exhibited frailty (defined as a score above 6 on the EFS) in the following groups: ESRD at 19% (*p* < 0.001), PD at 33% (*p* < 0.001), and KT at 16% (*p* = 0.004), compared to HS at 0%. Notably, hemodialysis patients had the highest number of patients with frailty, at 53% (*p* < 0.001, Figure 3a). Additionally, significant sex differences were observed among patients on HD, with 91% of women classified as frail compared to 38% of men (*p* = 0.012). When frailty was categorized into different levels (Figure 3b), patients on HD showed the highest percentage of severe frailty (a score over 10 on the EFS) at 9%, versus HS (0%, *p* < 0.001), ESRD (0%, *p* = 0.02), PD (6%, *p* = 0.04), and KT (0%, *p* = 0.01). Due to the small number of patients with severe and moderate frailty in the other groups, further subdivision of frailty levels was not performed for the remaining data analysis.

### 3.4. Frailty and Immune Cells in CKD Patients

Frailty was associated with distinct immune alterations across CKD treatment groups, as detailed in Supplementary Table S1. In ESRD patients, frail individuals tended to have lower T-helper cell counts (43 ± 8 vs. 49 ± 10; *p* = 0.08) and higher T-cytotoxic cell counts (31 ± 10 vs. 23 ± 9; *p* = 0.06) compared to fit patients, although these differences did not reach statistical significance. Conversely, the helper/cytotoxic (CD4/CD8) ratio was significantly lower in frail individuals (1.6 ± 0.7 vs. 2.5 ± 1.2; *p* = 0.03), indicating an imbalance in T-cell populations.

In the hemodialysis group, vulnerability to frailty was linked to significantly lower NK cell counts (105 ± 56 vs. 234 ± 99 in fit patients, *p* = 0.03) and a higher percentage of intermediate monocytes (20 ± 1% vs. 13 ± 6%, *p* = 0.09). Compared to frail patients (185 ± 70 NK cells; 10 ± 4% intermediate monocytes), vulnerable individuals showed intermediate values (*p* = 0.08 and *p* = 0.04, respectively). Additionally, frail patients had lower percentages of ICAM-1/CD54^+^ classical and non-classical monocytes (77 ± 10% and 81 ± 14%) compared to fit patients (87 ± 11%, *p* = 0.05; and 91 ± 8%, *p* = 0.09). CD54 expression levels were also reduced in frail individuals (classical: 145 ± 56 vs. 210 ± 82, *p* = 0.09; non-classical: 129 ± 61 vs. 199 ± 71, *p* = 0.02).

Among patients on peritoneal dialysis, frail individuals had lower T-cell counts compared to fit patients (786 ± 447 vs. 1163 ± 438, *p* = 0.04). The percentage of ICAM-1/CD54^+^ classical monocytes was also reduced in frail patients (84 ± 8%) compared to fit (91 ± 9%, *p* = 0.08) and vulnerable individuals (95 ± 7%, *p* = 0.04). Vulnerable patients exhibited higher frequencies of intermediate CD54^+^ (99 ± 1%) and classical CD86^+^ monocytes (92 ± 10%) than fit (*p* = 0.09 and *p* = 0.07, respectively) and frail patients (*p* = 0.004 and *p* = 0.06, respectively). Additionally, non-classical monocytes expressing CD86/B7.2 were more common in vulnerable than in fit patients (85 ± 20% vs. 71 ± 23%, *p* = 0.06), and their expression levels were significantly higher in frail individuals compared to vulnerable ones (209 ± 46 vs. 167 ± 89, *p* = 0.02).

In kidney transplant recipients, frail patients had higher T-cell (1607 ± 592) and T-cytotoxic cell counts (841 ± 409) compared to both fit (1147 ± 625, *p* = 0.09; 531 ± 313, *p* = 0.07) and vulnerable patients (1045 ± 327, *p* = 0.07; 380 ± 173, *p* = 0.01). Vulnerable patients showed lower B-cell counts (92 ± 43 vs. 147 ± 104 in fit patients, *p* = 0.06) and higher NK cell counts (296 ± 184 vs. 187 ± 152, *p* = 0.09). Finally, classical monocyte ICAM-1/CD54 expression (95 ± 8%) and intermediate monocyte CD86/B7.2 expression (98 ± 3%) were elevated in vulnerable patients compared to fit individuals (89 ± 9%, *p* = 0.07; and 94 ± 7%, *p* = 0.03, respectively).

### 3.5. Immuno-Fragile Profile

After confirming how frailty affects various immune parameters, we performed a principal component analysis (PCA) to identify a combined immuno-fragile profile by integrating key immune markers with frailty scores. The PCA identified three components with eigenvalues greater than 1. Sampling adequacy was considered appropriate (KMO = 0.695), and Bartlett’s test confirmed the data’s suitability for factor analysis (*p* < 0.001). Overall, these components explained 70% of the total variance, with Component 1 accounting for 38%, Component 2 for 19%, and Component 3 for 13%. As shown in Table 3, Component 1 was mainly characterized by lymphocyte subsets, Component 2 by monocyte subsets, and Component 3 by frailty indicators.

**Figure 4:**
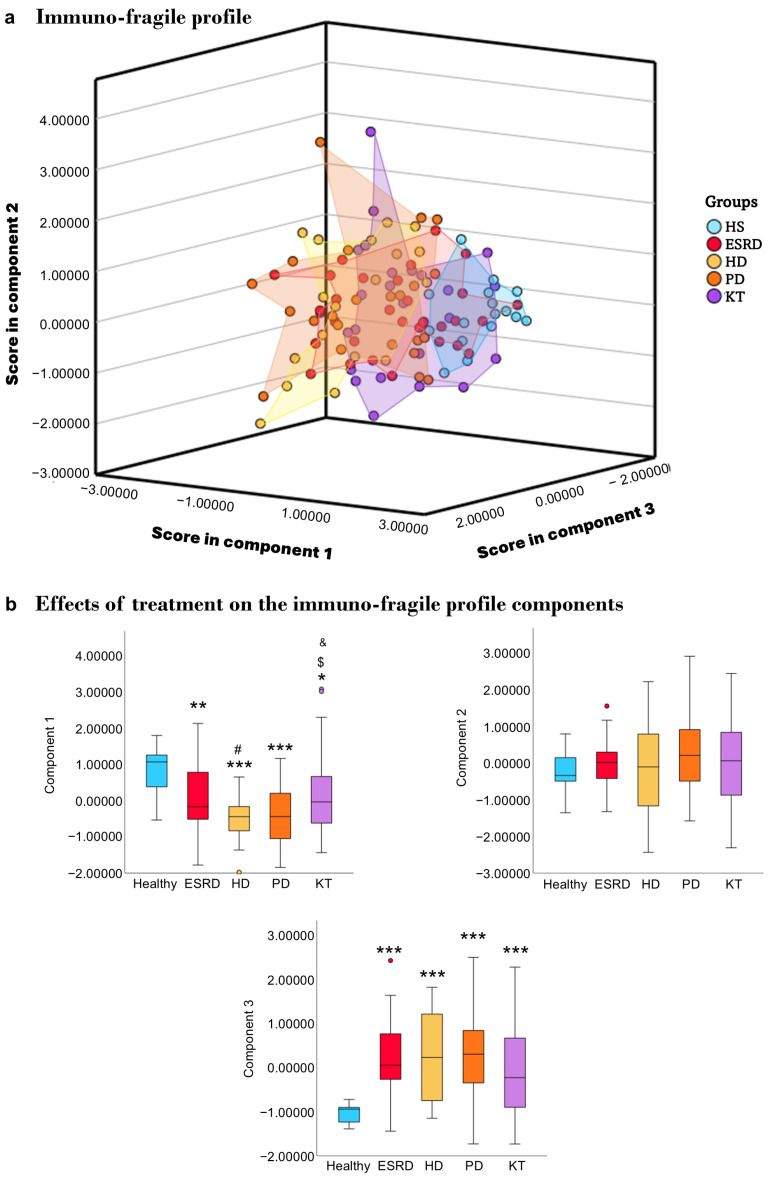
**Immuno-fragile profile.** Graphic representation of the immuno-fragile profile obtained from patients grouped according to their therapeutic group (**a**), and differences in each component among therapeutic groups (**b**). Principal component analyses sorted the immune variables into three components, accounting for 70% of the variance. The first component is primarily related to the number of lymphocytes (total, T, and B), the second to the expression of co-stimulatory molecules (CD54/ICAM-1 and CD86/B7.2) in monocytes, and the third to frailty. Abbreviations: HS, healthy subjects; ESRD, end-stage renal disease; HD, hemodialysis; PD, peritoneal dialysis; KT, kidney transplantation. Statistical significance was denoted by * *p* ≤ 0.05, ** *p* ≤ 0.01, *** *p* ≤ 0.001 vs. HS; ^#^ *p* ≤ 0.05 vs. ESRD; ^$^ *p* ≤ 0.05 vs. HD; ^&^ *p* ≤ 0.05 vs. PD.

The scores for each patient in each component were recorded, and a graph was created to clearly show the profiles of each patient group studied (Figure 4a). To verify that the patients indeed had distinct profiles, the score differences within each component for each group were analyzed (Figure 4b). Regarding component 1, where lymphocytes are the main participants, we observed that healthy individuals had different scores from patients with ESRD (*p* = 0.007), HD (*p* ≤ 0.001), PD (*p* ≤ 0.001), and KT (*p* = 0.013). Additionally, HD patients also showed differences in this component compared to ERCA (*p* = 0.025) and KT (*p* = 0.015), while DP patients differed from KT (*p* = 0.043). In component 2, where monocytes are primarily involved, no differences were observed between patients and healthy individuals. Finally, in the third component, where frailty plays a significant role, differences were noted between healthy individuals and patients regardless of treatment (*p* ≤ 0.001).

### 3.6. Survival

**Figure 5:**
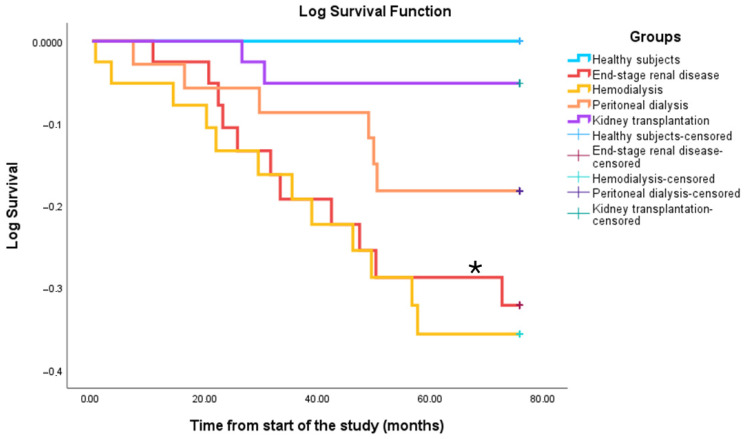
Comparison of survival curves among healthy individuals, patients with advanced chronic kidney disease, those on hemodialysis, peritoneal dialysis, and kidney transplant recipients. Statistical significance was denoted by * *p* ≤ 0.05.

**Figure 6:**
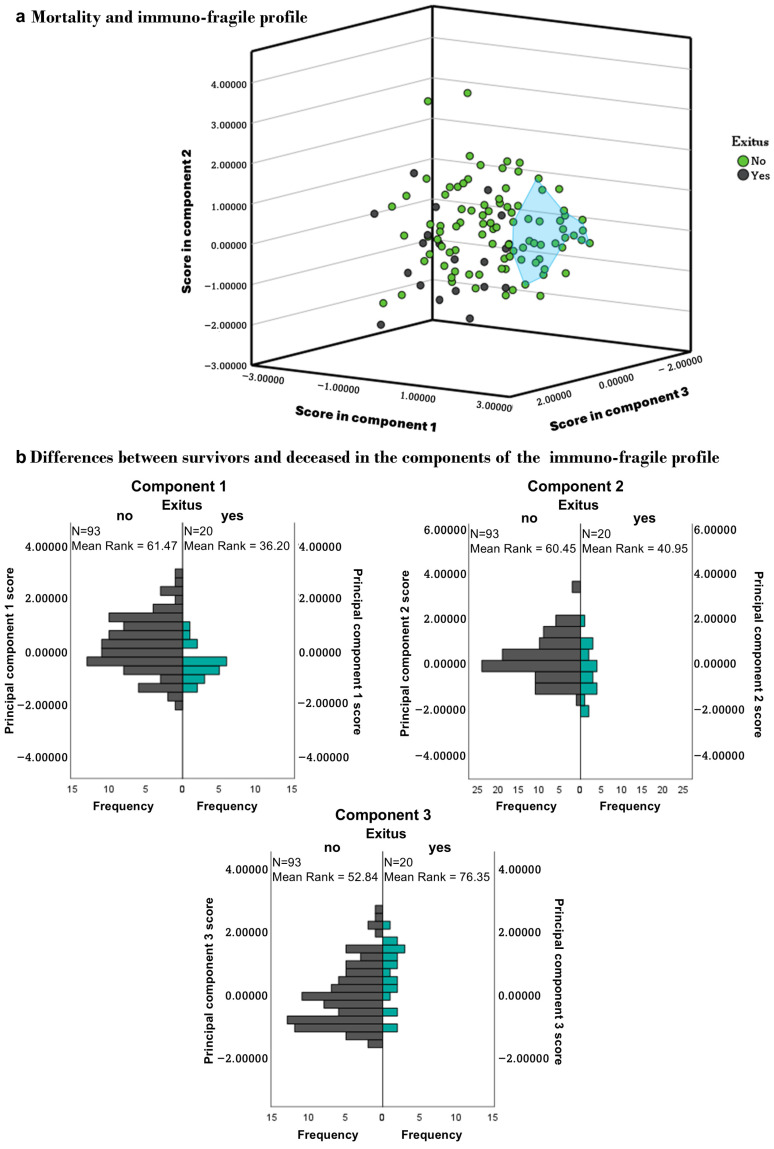
Differences between the immune-fragile survivors and deceased (exitus). The situation of deceased patients within the profile is compared with that of healthy controls, represented by the blue area (**a**), and the distribution of the latter across the components (**b**).

Between May 2018 and September 2024, 27 patients died: 11 with ESRD (37%), 12 on HD (40%), 2 on PD (7%), and 2 were KT recipients (5%). Survival rates were significantly lower in the HD (*p* = 0.01) and ESRD groups (*p* = 0.02) compared to healthy controls (Figure 5). Deceased patients had significantly lower counts of T, CD4, CD8, and B lymphocytes (*p* < 0.001). Component scores also differed between survivors and non-survivors: Component 1 (*p* = 0.002), Component 2 (*p* = 0.02), and Component 3 (*p* = 0.004) (Figure 6). A history of acute cerebrovascular accident (ACVA) was linked to higher mortality, particularly in HD patients (100% ACVA vs. 26% non-ACVA).

### 3.7. Validation of the Immunofragile Profile as a Prognostic Marker

To evaluate the prognostic value of the immuno-fragile profile, we conducted a multivariable Cox proportional hazards regression including the three PCA-derived components alongside established prognostic factors (age, sex, diabetes, and baseline cardiovascular disease). The overall model was significant (χ^2^ (7) = 40.0, *p* < 0.001). Higher scores in Component 1 (lymphocyte subsets) and Component 2 (monocyte subsets) were associated with a lower risk of death (HR = 0.502, 95% CI 0.27–0.93, *p* = 0.024; HR = 0.456, 95% CI 0.27–0.77, *p* = 0.004, respectively), whereas Component 3 (frailty indicators) was still associated with a higher risk of death (HR = 1.722, 95% CI 1.08–2.75, *p* = 0.023). Among the traditional risk factors, baseline cardiovascular disease showed the strongest link to mortality (HR = 13.744, 95% CI 2.56–73.7, *p* = 0.003). These findings show that the immuno-fragile profile offers prognostic information that is independent of established clinical factors predictors.

## 4. Discussion

In this cross-sectional study, we examined the phenotypic changes in lymphocytes and monocytes, the frailty levels of patients with chronic kidney disease (CKD), and the impact of varying degrees of frailty on immunity in patients with end-stage renal disease (ESRD), hemodialysis (HD), peritoneal dialysis (PD), and kidney transplantation (KT), compared to healthy individuals. Our findings indicate that patients with advanced CKD have an altered immune response and increased frailty, which correlates with higher mortality rates, especially among those on HD. This leads to distinct immuno-fragile profiles across different treatment modalities, which are important for understanding survival outcomes.

To our knowledge, this is the first study to evaluate CKD patients’ immune profiles and frail status across various therapeutic options within the same population. The immune system is a critical marker of health and longevity [43] and modulates aging. In chronic kidney disease (CKD), immunity plays a key role in the disease’s development and progression, as well as in adverse outcomes and the onset of comorbidities [13,14,15]. The build-up of uremic toxins contributes to immune dysfunction, chronic inflammation, and cellular senescence [44,45,46]. Despite advances in hemodialysis techniques, immune issues continue to persist, leading to increased morbidity and mortality among these patients [47].

Dialysis patients show lower total lymphocyte counts, including T lymphocytes and T-helper cells, with particularly notable reductions in those on hemodialysis. This condition is also associated with decreased T-cytotoxic cell counts and altered helper/cytotoxic ratios. Similar findings have been reported in other studies [48,49]. In ESRD, while some studies document lymphopenia [50], others report no significant differences [41]. In renal transplantation, lymphocyte levels typically remain stable except for changes in the helper/cytotoxic ratio, which aligns with our previous observations [41]. Regarding B cell counts, most studies indicate a decrease in B cells among CKD patients, regardless of treatment [22,51,52], consistent with our current findings. The decline in both lymphocyte types correlates with worsening renal function, loss of naïve T and B lymphocytes, and increased apoptosis caused by uremia [22,49,50,51,52,53]. These changes in lymphocyte profiles are linked to higher infection risk and poorer vaccine responses in CKD patients [16]. Additionally, B-lymphopenia has been associated with higher mortality rates among those on HD [22].

Intermediate and non-classical monocytes exhibit pro-inflammatory and atherogenic characteristics [14,26,54,55] and are found in increased numbers in hemodialysis patients [55,56], consistent with our results. The co-stimulatory molecule B7.2 plays a crucial role in mediating T-cell activation, and our study indicates that B7.2 expression is elevated in all patients examined. The existing literature presents conflicting findings; some studies report no significant changes [57], while others observe either an increase [41] or a decrease [58] in expression. ICAM-1, an adhesion molecule involved in the leukocyte synapse, showed increased expression solely in HD patients, aligning with prior reports [56]. Additionally, elevated B7.2 and ICAM-1 expression in CKD has been documented in intermediate and non-classical monocytes [41,59]. The upregulation of co-stimulatory molecules may reflect compensatory mechanisms in monocytes aimed at facilitating communication and activation of lymphocytes in response to previously noted alterations. Notably, increased ICAM-1 expression has been associated with enhanced activation of cytotoxic T-lymphocytes [60].

Notably, patients undergoing HD showed the highest level of immunosenescence, marked by lymphopenia, a lower CD4/CD8 ratio, and the expansion of pro-inflammatory monocyte subsets. Despite improvements in membrane biocompatibility, HD still exposes circulating cells to oxidative and antigenic stress and activates the complement system as blood flows through the dialyzer. These processes maintain chronic, low-grade inflammation (inflammaging) and accelerate immune aging, leading to a state of persistent immune dysfunction [61,62]. This provides a biological explanation for why patients with HD in our cohort exhibited the most severe immune impairment.

Frailty is a key indicator of biological aging. Several methods exist for assessing it, with the Edmonton Frail Scale validated for use even by non-geriatricians [42,63]. Studies consistently show that frailty increases as chronic kidney disease progresses and is especially common among patients on hemodialysis [64], as seen in our cohort. In this group, changes in bone and mineral metabolism along with muscle mass loss contribute to physical impairment, which is linked to cognitive decline, malnutrition, depression, reduced quality of life, cardiovascular and metabolic issues, and a higher risk of hospitalization, institutionalization, and death [64,65]. Additionally, oxidative and inflammatory stress in CKD has been directly connected to muscle dysfunction and greater frailty [66]. Importantly, our findings showed not only a higher prevalence of frailty in HD patients but also greater severity, with more individuals classified as moderately or severely frail. Recent prospective studies have confirmed that increased frailty severity in HD patients is associated with significantly higher mortality [67,68].

Although some research relates inflammation and oxidative stress, key factors in premature aging and frailty, the impact of frailty on the immune system remains scarce. A connection has been identified between frailty and proinflammatory cytokines, inadequate vaccine responses, and immune system impairments in frail patients [69,70,71,72]. Our study highlights notable variations in immune alterations among frail individuals; T lymphocyte populations and the expression of co-stimulatory molecules ICAM-1 and B7.2 in monocytes are generally compromised. Alterations in lymphocyte populations, particularly reducing T-helper and cytotoxic T cells [73], along with a proinflammatory monocyte phenotype [74], have been associated with frailty. Furthermore, interactions between these cell types are associated with increased osteoclast production, leading to an imbalance in bone metabolism [75]. Both sarcopenia and osteoporosis, closely related conditions, are characteristic of frailty [35,76]. Furthermore, recent studies show a high prevalence of sarcopenia in patients with CKD, especially those on dialysis [77].

Given the notable variability observed within each therapeutic group, we performed a principal component analysis to define an immune-fragile profile. This approach enables clear and straightforward monitoring of differences between the therapeutic groups and the healthy control population. Although external validation is needed, these profiles could aid in identifying patients at higher risk for comorbidities, as suggested for aging [43,78,79]. Some studies propose that immune profiles could serve as potential biomarkers for chronic kidney disease [48,80,81,82]. In this context, HD patients show the most significant variability and differences compared to the healthy population. These patients are not only prone to immunosenescence, inflammaging, and frailty [22,61,67,83,84] but also experience a high prevalence of chronic infectious diseases, cardiovascular problems, and increased mortality rates [85,86].

Our findings show a lower survival rate among patients undergoing hemodialysis (HD), consistent with existing literature. This decreased survival may result from significant immune alterations and increased frailty seen in this patient group [87,88,89]. Notably, patients who died had lymphopenia across all analyzed lymphocyte populations. Additionally, studies suggest a possible link between these immune changes and cardiovascular events [14], with higher mortality observed in patients with ACVA. Furthermore, the predictive ability of the immuno-fragile profile is supported by a Cox proportional hazards regression model, which confirms that the three components from the principal component analysis—lymphocytes, monocytes, and frailty—are independently associated with mortality risk. Higher scores in lymphocyte counts and co-stimulatory molecules in monocytes, which may indicate better immune competence and compensatory immune responses, are linked to lower mortality. In comparison, higher frailty scores correlate with increased risk. These results offer initial validation of the immuno-fragile profile as a potential clinical tool for both prognostic assessment and diagnostic stratification of CKD patients. However, larger and more diverse studies are necessary to confirm these findings, establish their broader applicability, and refine the profile’s definition. If validated, this composite immuno-fragile profile could become a valuable tool for identifying at-risk patients and guiding early, personalized intervention strategies.

## 5. Conclusions

In conclusion, this study provides strong evidence of premature immunosenescence and increased frailty in patients with chronic kidney disease, especially those on hemodialysis. Combining immune parameters and frailty scores through principal component analysis helped identify an immuno-fragile profile that not only distinguishes patient subgroups but also predicts survival outcomes. The independent association between this profile and mortality, as shown by Cox regression analysis, highlights its potential as both a diagnostic and prognostic tool in clinical practice.

While these findings are promising, they should be approached with caution. The small sample size, single-center design, and lack of internal and external validation of the PCA require further confirmation in larger and more diverse populations. Future research is necessary to confirm the consistency and clinical usefulness of the immuno-fragile profile. If verified, this method could aid in early risk detection and support personalized treatments designed to improve patients’ quality of life and outlook.

## Figures and Tables

**Table 1:** Demographic, clinical, and biochemical characteristics of the study population.

	HS	ESRD	HD	PD	KT
**Number of patients**	18	40	40	36	40
**Age (years), mean (range)**	51 (21–79)	61 ^a^ (22–82)	57 (21–84)	56 (24–82)	56 (25–80)
**Female, nº (%)**	9 (50%)	14 (35%)	13 (29%)	17 (47%)	15 (33%)
**Etiology of CKD nº (%)**					
**Diabetic nephropathy**	-	13 (33%)	4 (10%)	5 (14%)	8 (21%)
**Nephroangiosclerosis**	-	7 (17%)	6 (15%)	9 (26%)	6 (16%)
**Glomerular nephropathy**	-	6 (15%)	11 (28%)	11 (31%)	4 (11%)
**Interstitial nephritis**	-	6 (15%)	8 (20%)	4 (11%)	7 (18%)
**Polycystic kidney disease**	-	4 (10%)	1 (2%)	2 (6%)	8 (21%)
**Others**	-	4 (10%)	10 (25%)	4 (11%)	5 (13%)
**Body composition** **, mean (range)**					
**BMI**	24.6 (20.6–33.7)	27.4 ^a^ (15.7–41.1)	24.3 ^bb^ (17.3–36.4)	24.8 ^b^ (19.0–36.4)	27.0 ^c^ (17.0–39.5)
**Cardiovascular risk factors** **,** **nº (%)**					
**Hypertension**	1 (6%)	36 (90%) ^aaa^	33 (83%) ^aaa^	33 (92%) ^aaa^	39 (98%) ^aaac^
**Diabetes mellitus**	2 (11%)	18 (45%) ^a^	7 (18%) ^bb^	10 (28%)	15 (40%) ^ac^
**Dyslipidaemia**	0 (0%)	31 (78%) ^aaa^	23 (58%) ^aaa^	22 (61%) ^aaa^	21 (53%) ^aaab^
**Obesity (BMI ≥ 30 kg/m^2^)**	2 (11%)	10 (25%)	3 (8%) ^b^	4 (11%)	8 (24%)
**Smoking history**	4 (22%)	11 (28%)	15 (38%)	4 (11%) ^cc^	10 (25%)
**Treatment** **,** **nº (%)**					
**Statins**	0 (0)	30 (75%) ^aaa^	16 (40%) ^aabb^	20 (56%) ^aaa^	23 (58%) ^aaa^
**Allopurinol**	0 (0)	24 (60%) ^aaa^	8 (20%) ^abbb^	17 (47%) ^aaac^	11 (28%) ^abb^
**Antiplatelet**	0 (0)	8 (20%) ^a^	16 (40%) ^aa^	11 (31%) ^aa^	11 (28%) ^a^
**Erythropoietin**	0 (0)	19 (48%) ^aaa^	40 (100%) ^aaabbb^	20 (56%) ^aaaccc^	8 (21%) ^abbcccddd^

Abbreviations: HS, healthy subjects; ESRD, end-stage renal disease; HD, hemodialysis; PD, peritoneal dialysis; KT, kidney transplantation; CKD, chronic kidney disease; BMI, body mass index. Statistical significance was denoted by ^a^ *p* ≤ 0.05, ^aa^ *p* ≤ 0.01, ^aaa^ *p* ≤ 0.001 vs. HS; ^b^ *p* ≤ 0.05, ^bb^ *p* ≤ 0.01, ^bbb^ *p* ≤ 0.001 vs. ESRD; ^c^ *p* ≤ 0.05, ^cc^ *p* ≤ 0.01, ^ccc^ *p* ≤ 0.001 vs. HD; ^ddd^ *p* ≤ 0.001 vs. PD.

**Table 2:** Biochemical characteristics of the study population.

	HS	ESRD	HD	PD	KT
**eGFR (mL/min/1.73 m^2^), mean (range)**	>90	15 ^aaa^ (0–86)	16 ^aaa^ (0–66)	17 ^aaa^ (3–80)	47 ^bbbcccddd^ (8–81)
**Creatinine (mg/dL), mean (range)**	0.8 (0.6–1.1)	4.3 (2.4–7.8) ^aaa^	7.8 (2.8–2.5) ^aaabbb^	7.3 (4.0–5.3) ^aaabbb^	1.6 (0.9–3.9) ^abbbcccddd^
**Albumin (g/dL), mean (range)**	4.7 (4.1–5.0)	4.3 (3.6–5.0) ^aaa^	4.1 (3.3–4.8) ^aaa^	3.8 (2.6–4.5) ^aaabbbc^	4.5 (3.4–5.2) ^bbbcccddd^
**Proteins (mg/dL), mean (range)**	7.1 (6.5–8.0)	6.9 (5.5–7.9)	6.7 (5.2–8.6) ^aab^	6.5 (4.1–13.6) ^aaabbb^	7.0 (5.0–7.9) ^bbccddd^
**Uric acid (mg/dL), mean (range)**	5.0 (2.9–7.1)	6.5 ^aa^ (2.8–11.4)	5.8 (3.6–8.3)	5.7 (2.8–8.8)	6.9 ^aaacccddd^ (3.5–9.2)
**CRP (mg/dL), mean (range)**	0.3 (0.0–2.0)	0.5 ^aa^ (0.0–1.7)	0.9 ^aaa^ (0.0–12.8)	0.9 ^a^ (0.0–10.4)	0.5 ^a^ (0.0–5.5)

Abbreviations: HS, healthy subjects; ESRD, end-stage renal disease; HD, hemodialysis; PD, peritoneal dialysis; KT, kidney transplantation; eGFR, estimated glomerular filtration rate; CRP, C-reactive protein. Statistical significance was denoted by ^a^ *p* ≤ 0.05, ^aa^ *p* ≤ 0.01, ^aaa^ *p* ≤ 0.001 vs. HS; ^b^ *p* ≤ 0.05, ^bb^ *p* ≤ 0.01, ^bbb^ *p* ≤ 0.001 vs. ESRD; ^c^ *p* ≤ 0.05, ^cc^ *p* ≤ 0.01, ^ccc^ *p* ≤ 0.001 vs. HD; ^ddd^ *p* ≤ 0.001 vs. PD.

**Table 3:** Immunity and frailty variables used in developing the immuno-fragile profile. The contribution of these variables to each component is noted.

	Component 1	Component 2	Component 3
**Frailty (score)**	−0.242	0.003	0.927
**Total lymphocytes (nº)**	0.924	0.196	0.123
**T lymphocytes (nº)**	0.858	0.210	0.197
**B lymphocytes (nº)**	0.554	0.165	−0.137
**Intermediate monocytes (%)**	−0.201	0.619	0.004
**Amount of B7.2/CD86 expressed by non-classical monocytes**	−0.326	0.733	−0.276
**Amount of ICAM-1/CD54 expressed by non-classical monocytes**	0.362	0.694	0.213

## Data Availability

The data supporting the findings of this study are available from the corresponding author upon reasonable request.

## Supplementary Materials

The following supporting information can be downloaded at https://www.mdpi.com/article/10.3390/biomedicines13102370/s1, Figure S1: Overview of the study design and participants; Figure S2: Rate of expression of co-stimulatory molecules in monocytes; Table S1: Effect of frailty on the immune cells in end-stage renal disease, hemodialysis, peritoneal dialysis, and kidney transplantation patients.

## Abbreviations

The following abbreviations are used in this manuscript:

ACVA	Acute cerebrovascular accident
BMI	Body mass index
CD	Cluster of differentiation
CKD	Chronic kidney disease
CRP	C-reactive protein
EFS	Edmonton Frail Scale
eGFR	Estimated glomerular filtration rate
ESRD	End-stage renal disease
HD	Hemodialysis
HR	Hazard ratios
HS	Healthy subjects
ICAM-1	Intercellular adhesion molecule 1
IS	Immune system
KT	Kidney transplant patients
PCA	Principal component analysis
PD	Peritoneal dialysis

## References

[1] Francis A., Harhay M.N., Ong A.C.M., Tummalapalli S.L., Ortiz A., Fogo A.B., Fliser D., Roy-Chaudhury P., Fontana M., Nangaku M. (2024). Chronic Kidney Disease and the Global Public Health Agenda: An International Consensus. Nat. Rev. Nephrol..

[2] Ying M., Shao X., Qin H., Yin P., Lin Y., Wu J., Ren J., Zheng Y. (2024). Disease Burden and Epidemiological Trends of Chronic Kidney Disease at the Global, Regional, National Levels from 1990 to 2019. Nephron.

[3] Kovesdy C.P. (2022). Epidemiology of Chronic Kidney Disease: An Update 2022. Kidney Int. Suppl. (2011).

[4] Tang Y., Jiang J., Zhao Y., Du D. (2024). Aging and Chronic Kidney Disease: Epidemiology, Therapy, Management and the Role of Immunity. Clin. Kidney J..

[5] Chesnaye N.C., Ortiz A., Zoccali C., Stel V.S., Jager K.J. (2024). The Impact of Population Ageing on the Burden of Chronic Kidney Disease. Nat. Rev. Nephrol..

[6] He Y., Tang W., Chen J., Tang J., Zheng Y., Wang X., Xing B., Li X., Xu Y., Wang X. (2025). Global Burden of Chronic Kidney Disease Due to Hypertension (1990–2021): A Systematic Analysis of Epidemiological Trends, Risk Factors, and Projections to 2036 from the Gbd 2021 Study. BMC Nephrol..

[7] Bikbov B., Purcell C.A., Levey A.S., Smith M., Abdoli A., Abebe M., Adebayo O.M., Afarideh M., Agarwal S.K., Agudelo-Botero M. (2020). Global, Regional, and National Burden of Chronic Kidney Disease, 1990–2017: A Systematic Analysis for the Global Burden of Disease Study 2017. Lancet.

[8] Lv J.C., Zhang L.X. (2019). Prevalence and Disease Burden of Chronic Kidney Disease. Adv. Exp. Med. Biol..

[9] Zhang Y., Yu C., Li X. (2024). Kidney Aging and Chronic Kidney Disease. Int. J. Mol. Sci..

[10] Ebert T., Pawelzik S.C., Witasp A., Arefin S., Hobson S., Kublickiene K., Shiels P.G., Back M., Stenvinkel P. (2020). Inflammation and Premature Ageing in Chronic Kidney Disease. Toxins.

[11] Yamamoto T., Isaka Y. (2024). Pathological Mechanisms of Kidney Disease in Ageing. Nat. Rev. Nephrol..

[12] Espi M., Koppe L., Fouque D., Thaunat O. (2020). Chronic Kidney Disease-Associated Immune Dysfunctions: Impact of Protein-Bound Uremic Retention Solutes on Immune Cells. Toxins.

[13] Kooman J.P., Dekker M.J., Usvyat L.A., Kotanko P., van der Sande F.M., Schalkwijk C.G., Shiels P.G., Stenvinkel P. (2017). Inflammation and Premature Aging in Advanced Chronic Kidney Disease. Am. J. Physiol. Ren. Physiol..

[14] Carracedo J., Alique M., Vida C., Bodega G., Ceprian N., Morales E., Praga M., de Sequera P., Ramirez R. (2020). Mechanisms of Cardiovascular Disorders in Patients with Chronic Kidney Disease: A Process Related to Accelerated Senescence. Front. Cell Dev. Biol..

[15] Crepin T., Legendre M., Carron C., Vachey C., Courivaud C., Rebibou J.M., Ferrand C., Laheurte C., Vauchy C., Gaiffe E. (2020). Uraemia-Induced Immune Senescence and Clinical Outcomes in Chronic Kidney Disease Patients. Nephrol. Dial. Transplant..

[16] Syed-Ahmed M., Narayanan M. (2019). Immune Dysfunction and Risk of Infection in Chronic Kidney Disease. Adv. Chronic Kidney Dis..

[17] Stenvinkel P., Larsson T.E. (2013). Chronic Kidney Disease: A Clinical Model of Premature Aging. Am. J. Kidney Dis..

[18] Claro L.M., Moreno-Amaral A.N., Gadotti A.C., Dolenga C.J., Nakao L.S., Azevedo M.L.V., de Noronha L., Olandoski M., de Moraes T.P., Stinghen A.E.M. (2018). The Impact of Uremic Toxicity Induced Inflammatory Response on the Cardiovascular Burden in Chronic Kidney Disease. Toxins.

[19] Webster A.C., Nagler E.V., Morton R.L., Masson P. (2017). Chronic Kidney Disease. Lancet.

[20] Lee T.H., Chen J.J., Wu C.Y., Lin T.Y., Hung S.C., Yang H.Y. (2024). Immunosenescence, Gut Dysbiosis, and Chronic Kidney Disease: Interplay and Implications for Clinical Management. Biomed. J..

[21] Cozzolino M., Magagnoli L., Ciceri P. (2025). From Physicochemical Classification to Multidimensional Insights: A Comprehensive Review of Uremic Toxin Research. Toxins.

[22] Molina M., Allende L.M., Ramos L.E., Gutierrez E., Pleguezuelo D.E., Hernandez E.R., Rios F., Fernandez C., Praga M., Morales E. (2018). CD19(+) B-Cells, a New Biomarker of Mortality in Hemodialysis Patients. Front. Immunol..

[23] Betjes M.G., Litjens N.H. (2015). Chronic Kidney Disease and Premature Ageing of the Adaptive Immune Response. Curr. Urol. Rep..

[24] Mihai S., Codrici E., Popescu I.D., Enciu A.M., Albulescu L., Necula L.G., Mambet C., Anton G., Tanase C. (2018). Inflammation-Related Mechanisms in Chronic Kidney Disease Prediction, Progression, and Outcome. J. Immunol. Res..

[25] Ducloux D., Legendre M., Bamoulid J., Saas P., Courivaud C., Crepin T. (2021). End-Stage Renal Disease-Related Accelerated Immune Senescence: Is Rejuvenation of the Immune System a Therapeutic Goal?. Front. Med..

[26] Vaziri N.D., Pahl M.V., Crum A., Norris K. (2012). Effect of Uremia on Structure and Function of Immune System. J. Ren. Nutr..

[27] Foresto-Neto O., Menezes-Silva L., Leite J.A., Andrade-Silva M., Camara N.O.S. (2024). Immunology of Kidney Disease. Annu. Rev. Immunol..

[28] Hu M., Wang Q., Liu B., Ma Q., Zhang T., Huang T., Lv Z., Wang R. (2022). Chronic Kidney Disease and Cancer: Inter-Relationships and Mechanisms. Front. Cell Dev. Biol..

[29] Alvarez-Lara M.A., Carracedo J., Ramirez R., Martin-Malo A., Rodriguez M., Madueno J.A., Aljama P. (2004). The Imbalance in the Ratio of Th1 and Th2 Helper Lymphocytes in Uraemia Is Mediated by an Increased Apoptosis of Th1 Subset. Nephrol. Dial. Transplant..

[30] Dounousi E., Duni A., Naka K.K., Vartholomatos G., Zoccali C. (2021). The Innate Immune System and Cardiovascular Disease in Eskd: Monocytes and Natural Killer Cells. Curr. Vasc. Pharmacol..

[31] Valera G., Figuer A., Caro J., Yuste C., Morales E., Ceprian N., Bodega G., Ramirez R., Alique M., Carracedo J. (2023). Plasma Glycocalyx Pattern: A Mirror of Endothelial Damage in Chronic Kidney Disease. Clin. Kidney J..

[32] Bernelot Moens S.J., Verweij S.L., van der Valk F.M., van Capelleveen J.C., Kroon J., Versloot M., Verberne H.J., Marquering H.A., Duivenvoorden R., Vogt L. (2017). Arterial and Cellular Inflammation in Patients with Ckd. J. Am. Soc. Nephrol..

[33] Heine G.H., Ortiz A., Massy Z.A., Lindholm B., Wiecek A., Martinez-Castelao A., Covic A., Goldsmith D., Suleymanlar G., London G.M. (2012). Monocyte Subpopulations and Cardiovascular Risk in Chronic Kidney Disease. Nat. Rev. Nephrol..

[34] Roshanravan B., Khatri M., Robinson-Cohen C., Levin G., Patel K.V., de Boer I.H., Seliger S., Ruzinski J., Himmelfarb J., Kestenbaum B. (2012). A Prospective Study of Frailty in Nephrology-Referred Patients with Ckd. Am. J. Kidney Dis..

[35] Fried L.P., Tangen C.M., Walston J., Newman A.B., Hirsch C., Gottdiener J., Seeman T., Tracy R., Kop W.J., Burke G. (2001). Frailty in Older Adults: Evidence for a Phenotype. J. Gerontol. A Biol. Sci. Med. Sci..

[36] Cawthon P.M., Marshall L.M., Michael Y., Dam T.T., Ensrud K.E., Barrett-Connor E., Orwoll E.S., Osteoporotic Fractures in Men Research G. (2007). Frailty in Older Men: Prevalence, Progression, and Relationship with Mortality. J. Am. Geriatr. Soc..

[37] Kojima G., Liljas A.E.M., Iliffe S. (2019). Frailty Syndrome: Implications and Challenges for Health Care Policy. Risk Manag. Healthc. Policy.

[38] Pereira A., Midao L., Almada M., Costa E. (2021). Pre-Frailty and Frailty in Dialysis and Pre-Dialysis Patients: A Systematic Review of Clinical and Biochemical Markers. Int. J. Environ. Res. Public Health.

[39] Ballew S.H., Chen Y., Daya N.R., Godino J.G., Windham B.G., McAdams-DeMarco M., Coresh J., Selvin E., Grams M.E. (2017). Frailty, Kidney Function, and Polypharmacy: The Atherosclerosis Risk in Communities (Aric) Study. Am. J. Kidney Dis..

[40] Walker S.R., Brar R., Eng F., Komenda P., Rigatto C., Prasad B., Bohm C.J., Storsley L.J., Tangri N. (2015). Frailty and Physical Function in Chronic Kidney Disease: The Canfit Study. Can. J. Kidney Health Dis..

[41] Ceprian N., Valera G., Caro J., Yuste C., Serroukh N., Gonzalez de Pablos I., Oliva C., Figuer A., Praga M., Alique M. (2021). Effect of Kidney Transplantation on Accelerated Immunosenescence and Vascular Changes Induced by Chronic Kidney Disease. Front. Med..

[42] Rolfson D.B., Majumdar S.R., Tsuyuki R.T., Tahir A., Rockwood K. (2006). Validity and Reliability of the Edmonton Frail Scale. Age Ageing.

[43] Martínez de Toda I., Ceprián N., Díaz-Del Cerro E., De la Fuente M. (2021). The Role of Immune Cells in Oxi-Inflamm-Aging. Cells.

[44] Sanchez-Ospina D., Mas-Fontao S., Gracia-Iguacel C., Avello A., Gonzalez de Rivera M., Mujika-Marticorena M., Gonzalez-Parra E. (2024). Displacing the Burden: A Review of Protein-Bound Uremic Toxin Clearance Strategies in Chronic Kidney Disease. J. Clin. Med..

[45] Ramírez R., Ceprian N., Figuer A., Valera G., Bodega G., Alique M., Carracedo J. (2022). Endothelial Senescence and the Chronic Vascular Diseases: Challenges and Therapeutic Opportunities in Atherosclerosis. J. Pers. Med..

[46] Cohen G. (2020). Immune Dysfunction in Uremia 2020. Toxins.

[47] Campo S., Lacquaniti A., Trombetta D., Smeriglio A., Monardo P. (2022). Immune System Dysfunction and Inflammation in Hemodialysis Patients: Two Sides of the Same Coin. J. Clin. Med..

[48] Gonzalez-Cuadrado C., Caro-Espada P.J., Chivite-Lacaba M., Utrero-Rico A., Lozano-Yuste C., Gutierrez-Solis E., Morales E., Sandino-Perez J., Gil-Etayo F.J., Allende-Martinez L. (2023). Hemodialysis-Associated Immune Dysregulation in SARS-CoV-2-Infected End-Stage Renal Disease Patients. Int. J. Mol. Sci..

[49] Borges A., Borges M., Fernandes J., Nascimento H., Sameiro-Faria M., Miranda V., Reis F., Belo L., Costa E., Santos-Silva A. (2011). Apoptosis of Peripheral Cd4(+) T-Lymphocytes in End-Stage Renal Disease Patients under Hemodialysis and Rhepo Therapies. Ren. Fail..

[50] Xiang F.F., Zhu J.M., Cao X.S., Shen B., Zou J.Z., Liu Z.H., Zhang H., Teng J., Liu H., Ding X.Q. (2016). Lymphocyte Depletion and Subset Alteration Correlate to Renal Function in Chronic Kidney Disease Patients. Ren. Fail..

[51] Moser B., Roth G., Brunner M., Lilaj T., Deicher R., Wolner E., Kovarik J., Boltz-Nitulescu G., Vychytil A., Ankersmit H.J. (2003). Aberrant T Cell Activation and Heightened Apoptotic Turnover in End-Stage Renal Failure Patients: A Comparative Evaluation between Non-Dialysis, Haemodialysis, and Peritoneal Dialysis. Biochem. Biophys. Res. Commun..

[52] Fernandez-Fresnedo G., Ramos M.A., Gonzalez-Pardo M.C., de Francisco A.L., Lopez-Hoyos M., Arias M. (2000). B Lymphopenia in Uremia Is Related to an Accelerated in Vitro Apoptosis and Dysregulation of Bcl-2. Nephrol. Dial. Transplant..

[53] Litjens N.H., van Druningen C.J., Betjes M.G. (2006). Progressive Loss of Renal Function Is Associated with Activation and Depletion of Naive T Lymphocytes. Clin. Immunol..

[54] Merino A., Buendia P., Martin-Malo A., Aljama P., Ramirez R., Carracedo J. (2011). Senescent Cd14+Cd16+ Monocytes Exhibit Proinflammatory and Proatherosclerotic Activity. J. Immunol..

[55] Heine G.H., Ulrich C., Seibert E., Seiler S., Marell J., Reichart B., Krause M., Schlitt A., Kohler H., Girndt M. (2008). Cd14(++)Cd16+ Monocytes but Not Total Monocyte Numbers Predict Cardiovascular Events in Dialysis Patients. Kidney Int..

[56] Ramirez R., Carracedo J., Berdud I., Carretero D., Merino A., Rodriguez M., Tetta C., Martin-Malo A., Aljama P. (2006). Microinflammation in Hemodialysis Is Related to a Preactivated Subset of Monocytes. Hemodial. Int..

[57] Girndt M., Sester M., Sester U., Kaul H., Kohler H. (2001). Defective Expression of B7-2 (Cd86) on Monocytes of Dialysis Patients Correlates to the Uremia-Associated Immune Defect. Kidney Int..

[58] Verkade M.A., van Druningen C.J., Vaessen L.M., Hesselink D.A., Weimar W., Betjes M.G. (2007). Functional Impairment of Monocyte-Derived Dendritic Cells in Patients with Severe Chronic Kidney Disease. Nephrol. Dial. Transplant..

[59] Wong K.L., Yeap W.H., Tai J.J., Ong S.M., Dang T.M., Wong S.C. (2012). The Three Human Monocyte Subsets: Implications for Health and Disease. Immunol. Res..

[60] Goldstein J.S., Chen T., Gubina E., Pastor R.W., Kozlowski S. (2000). Icam-1 Enhances Mhc-Peptide Activation of Cd8(+) T Cells without an Organized Immunological Synapse. Eur. J. Immunol..

[61] Petho A.G., Fulop T., Orosz P., Szenasi G., Tapolyai M., Dezsi L. (2025). Increased Cardiovascular Mortality in Hemodialysis: The Role of Chronic Inflammation, Complement Activation, and Non-Biocompatibility. Toxins.

[62] Song Z., Tsou S., Martin F., Kayumov M., Xiao Y., Zhou H., Abdi R., Tullius S.G. (2025). Kidney Disease as a Driver of Immunosenescence: Mechanisms and Potential Interventions. J. Am. Soc. Nephrol..

[63] Perna S., Francis M.D., Bologna C., Moncaglieri F., Riva A., Morazzoni P., Allegrini P., Isu A., Vigo B., Guerriero F. (2017). Performance of Edmonton Frail Scale on Frailty Assessment: Its Association with Multi-Dimensional Geriatric Conditions Assessed with Specific Screening Tools. BMC Geriatr..

[64] Chowdhury R., Peel N.M., Krosch M., Hubbard R.E. (2017). Frailty and Chronic Kidney Disease: A Systematic Review. Arch. Gerontol. Geriatr..

[65] Kennard A., Glasgow N., Rainsford S., Talaulikar G. (2023). Frailty in Chronic Kidney Disease: Challenges in Nephrology Practice. A Review of Current Literature. Intern. Med. J..

[66] Wesson D.E., Mathur V., Tangri N. (2022). Metabolic Basis and Pathogenesis of Skeletal Muscle Dysfunction as Cause of Frailty in Chronic Kidney Disease. Am. J. Nephrol..

[67] Tonelli M., Wiebe N., Gill J.S., Bello A.K., Hemmelgarn B.R., Chan C.T., Lloyd A., Thadhani R.I., Thompson S. (2023). Frailty and Clinical Outcomes in Patients Treated with Hemodialysis: A Prospective Cohort Study. Kidney Med..

[68] Fu W., Zhang A., Ma L., Jia L., Chhetri J.K., Chan P. (2021). Severity of Frailty as a Significant Predictor of Mortality for Hemodialysis Patients: A Prospective Study in China. Int. J. Med. Sci..

[69] Hubbard R.E., O’Mahony M.S., Savva G.M., Calver B.L., Woodhouse K.W. (2009). Inflammation and Frailty Measures in Older People. J. Cell. Mol. Med..

[70] Yao X., Hamilton R.G., Weng N.P., Xue Q.L., Bream J.H., Li H., Tian J., Yeh S.H., Resnick B., Xu X. (2011). Frailty Is Associated with Impairment of Vaccine-Induced Antibody Response and Increase in Post-Vaccination Influenza Infection in Community-Dwelling Older Adults. Vaccine.

[71] Buondonno I., Sassi F., Cattaneo F., D’Amelio P. (2022). Association between Immunosenescence, Mitochondrial Dysfunction and Frailty Syndrome in Older Adults. Cells.

[72] De Maeyer R.P.H., Akbar A.N. (2022). Aging and frailty immune landscape. Nat. Aging.

[73] Navarro-Martinez R., Cauli O. (2021). Lymphocytes as a Biomarker of Frailty Syndrome: A Scoping Review. Diseases.

[74] Cybularz M., Wydra S., Berndt K., Poitz D.M., Barthel P., Alkouri A., Heidrich F.M., Ibrahim K., Jellinghaus S., Speiser U. (2021). Frailty Is Associated with Chronic Inflammation and Pro-Inflammatory Monocyte Subpopulations. Exp. Gerontol..

[75] Cafiero C., Gigante M., Brunetti G., Simone S., Chaoul N., Oranger A., Ranieri E., Colucci S., Pertosa G.B., Grano M. (2018). Inflammation Induces Osteoclast Differentiation from Peripheral Mononuclear Cells in Chronic Kidney Disease Patients: Crosstalk between the Immune and Bone Systems. Nephrol. Dial. Transplant..

[76] Greco E.A., Pietschmann P., Migliaccio S. (2019). Osteoporosis and Sarcopenia Increase Frailty Syndrome in the Elderly. Front. Endocrinol..

[77] Duarte M.P., Almeida L.S., Neri S.G.R., Oliveira J.S., Wilkinson T.J., Ribeiro H.S., Lima R.M. (2024). Prevalence of Sarcopenia in Patients with Chronic Kidney Disease: A Global Systematic Review and Meta-Analysis. J. Cachexia Sarcopenia Muscle.

[78] Martinez de Toda I., Vida C., Diaz-Del Cerro E., De la Fuente M. (2021). The Immunity Clock. J. Gerontol. A Biol. Sci. Med. Sci..

[79] Alpert A., Pickman Y., Leipold M., Rosenberg-Hasson Y., Ji X., Gaujoux R., Rabani H., Starosvetsky E., Kveler K., Schaffert S. (2019). A Clinically Meaningful Metric of Immune Age Derived from High-Dimensional Longitudinal Monitoring. Nat. Med..

[80] Crepin T., Gaiffe E., Courivaud C., Roubiou C., Laheurte C., Moulin B., Frimat L., Rieu P., Mousson C., Durrbach A. (2016). Pre-Transplant End-Stage Renal Disease-Related Immune Risk Profile in Kidney Transplant Recipients Predicts Post-Transplant Infections. Transpl. Infect. Dis..

[81] Zaza G., Granata S., Rascio F., Pontrelli P., Dell’Oglio M.P., Cox S.N., Pertosa G., Grandaliano G., Lupo A. (2013). A Specific Immune Transcriptomic Profile Discriminates Chronic Kidney Disease Patients in Predialysis from Hemodialyzed Patients. BMC Med. Genom..

[82] Kim K.W., Chung B.H., Jeon E.J., Kim B.M., Choi B.S., Park C.W., Kim Y.S., Cho S.G., Cho M.L., Yang C.W. (2012). B Cell-Associated Immune Profiles in Patients with End-Stage Renal Disease (Esrd). Exp. Mol. Med..

[83] Sasaki K., Shoji T., Kabata D., Shintani A., Okute Y., Tsuchikura S., Shimomura N., Tsujimoto Y., Nakatani S., Mori K. (2021). Oxidative Stress and Inflammation as Predictors of Mortality and Cardiovascular Events in Hemodialysis Patients: The DREAM Cohort. J. Atheroscler. Thromb..

[84] Zimmermann J., Herrlinger S., Pruy A., Metzger T., Wanner C. (1999). Inflammation Enhances Cardiovascular Risk and Mortality in Hemodialysis Patients. Kidney Int..

[85] Greeviroj P., Lertussavavivat T., Thongsricome T., Takkavatakarn K., Phannajit J., Avihingsanon Y., Praditpornsilpa K., Eiam-Ong S., Susantitaphong P. (2022). The World Prevalence, Associated Risk Factors and Mortality of Hepatitis C Virus Infection in Hemodialysis Patients: A Meta-Analysis. J. Nephrol..

[86] Sinnakirouchenan R., Holley J.L. (2011). Peritoneal Dialysis Versus Hemodialysis: Risks, Benefits, and Access Issues. Adv. Chronic Kidney Dis..

[87] Wang Y., Zhou Y., Huang C., Wang Y., Lou L., Zhao L., Xu S., Zheng M., Li S. (2025). Development of a Prediction Model for in-Hospital Mortality in Immunocompromised Chronic Kidney Diseases Patients with Severe Infection. BMC Nephrol..

[88] Kennard A., Richardson A., Rainsford S., Hamilton K., Glasgow N., Pumpa K., Douglas A., Talaulikar G.S. (2024). Longitudinal Frailty Assessment in the Prediction of Survival Among Patients with Advanced Chronic Kidney Disease: A Prospective Observational Single-Centre Cohort Study. BMJ Open.

[89] Hannan M., Chen J., Hsu J., Zhang X., Saunders M.R., Brown J., McAdams-DeMarco M., Mohanty M.J., Vyas R., Hajjiri Z. (2024). Frailty and Cardiovascular Outcomes in Adults with Ckd: Findings from the Chronic Renal Insufficiency Cohort (Cric) Study. Am. J. Kidney Dis..

