# Supplementary material for: An Immuno-Fragile Profile Is Associated with Mortality Risk in Patients with Chronic Kidney Disease
Source: Biomedicines. 2025 Sep 27;13(10):2370. doi: 10.3390/biomedicines13102370 (PMC12561049; doi:10.3390/biomedicines13102370)
Supplement: Supplementary file 1 [file biomedicines-13-02370-s001.zip › biomedicines-3855878-supplementary.pdf]

An Immuno-Fragile Profile Is Associated with Mortality Risk in Patients with Chronic Kidney Disease

Noemí Ceprián [1,2,*], Irene Martínez de Toda [1,2,*], Paula Jara Caro [2,3], Claudia Yuste [2,3], Gemma Valera-Arévalo [1,2], Ignacio González de Pablos [3], Andrea Figuer [4,5], Matilde Alique [4,5], Rafael Ramírez [4,5], Enrique Morales [2,3,6,†] and Julia Carracedo [1,2,†]

[1] Departamento de Genética, Fisiología y Microbiología, Facultad de Ciencias Biológicas, Universidad Complutense de Madrid, 28040 Madrid, Spain. gvalera@ucm.es (G.V.-A.); julcar01@ucm.es (J.C.)
[2] Instituto de Investigación Sanitaria Hospital 12 de Octubre (imas12), RICORS 2040, 28041 Madrid, Spain
[3] Departamento de Nefrología, Hospital Universitario 12 de Octubre, RICORS 2040, 28041 Madrid, Spain; jcaroespada@gmail.com (P.J.C.); claudiayustelozano@yahoo.es (C.Y.); igp.snurse@gmail.com (I.G.P.); emoralesr@senefro.org (E.M.)
[4] Departamento de Biología de Sistemas, Universidad de Alcalá, 28871 Alcalá de Henares, Spain; andrea.figuer@salud.madrid.org (A.F.); matilde.alique@uah.es (M.A.); manuel.ramirez@uah.es (R.R.)
[5] Instituto Ramón y Cajal de Investigación Sanitaria (IRYCIS), 28034, Madrid, Spain
[6] Departamento de Medicina, Facultad de Medicina, Universidad Complutense de Madrid, 28040 Madrid, Spain
* Correspondence: nceprian@ucm.es (N.C.); imtcabeza@ucm.es (I.M.d.T.)
† These authors contributed equally to this work.

SUPPLEMENTARY MATERIAL

Supplementary Figures

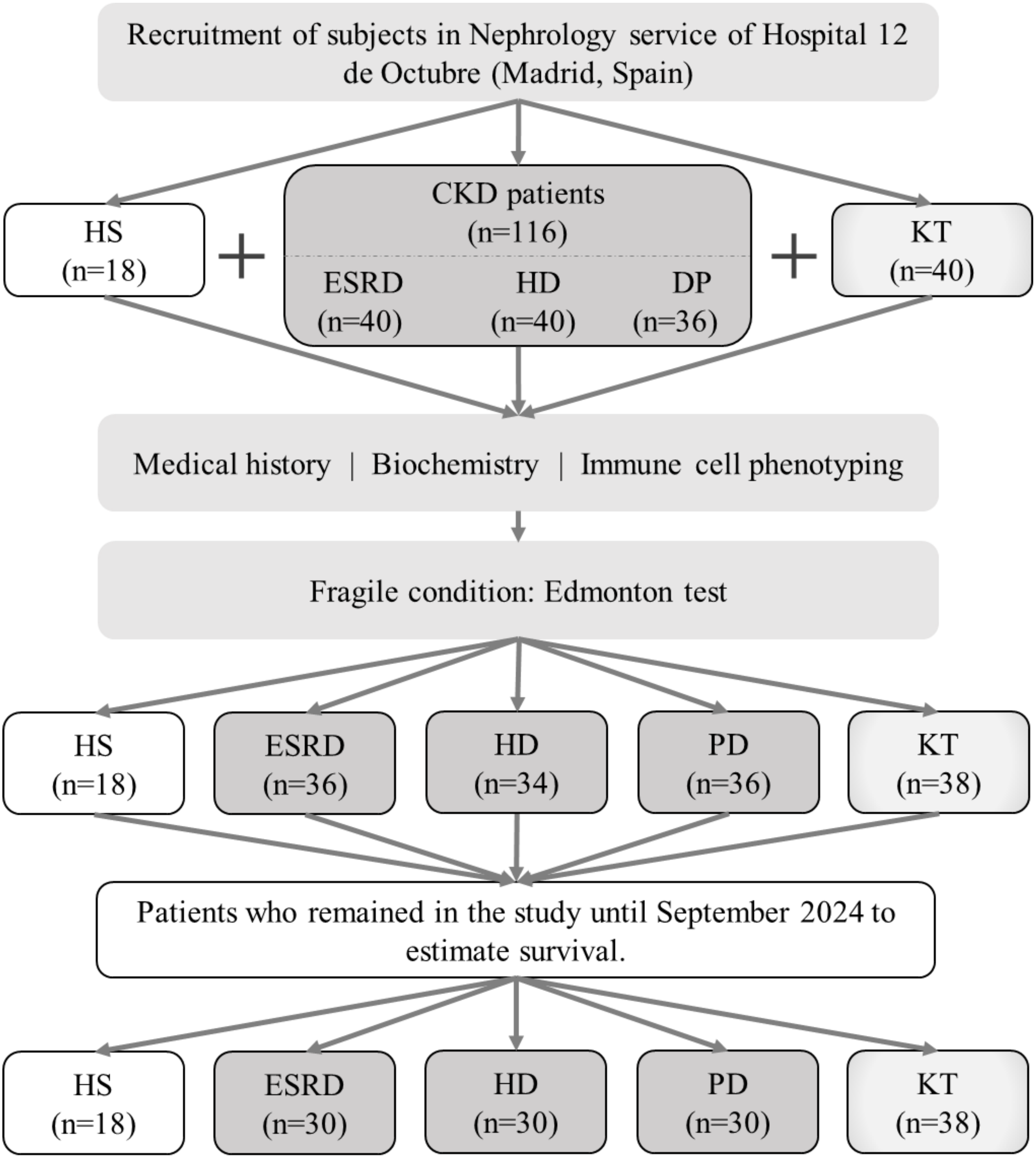

Figure S1. Overview of the study design and participants. CKD: chronic kidney disease; ESRD: end-stage renal disease; HD: hemodialysis; PD: peritoneal dialysis; KT: kidney transplantation.

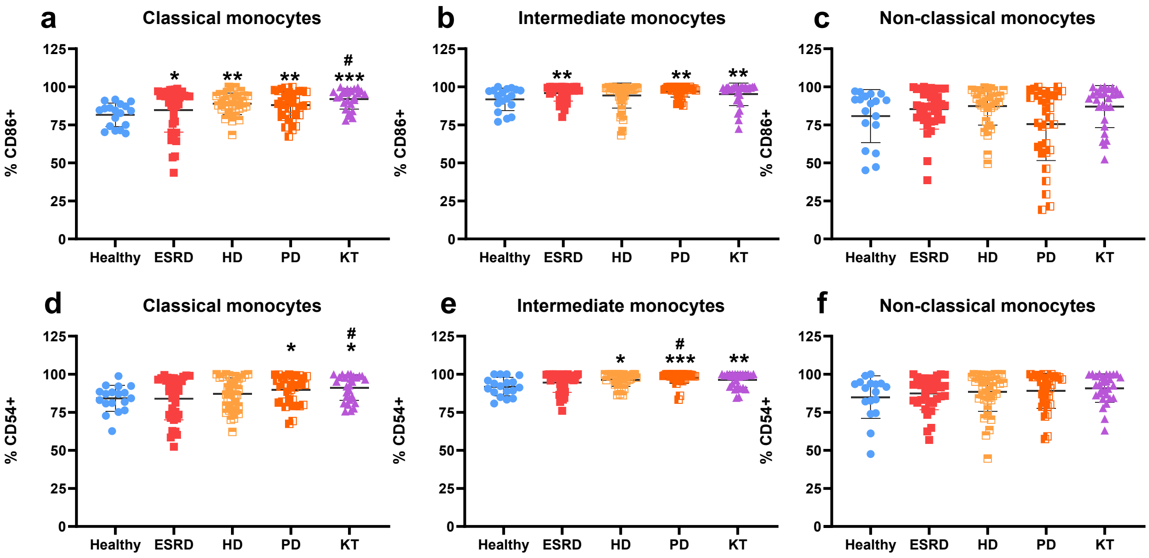

o

Figure S2. **Rate of expression of co-stimulatory molecules in monocytes**. Percentage of classical **(a)**, intermediate **(b)**, and non-classical **(c)** monocytes expressing B7.1/CD86 expressed, and percentage of classical **(d)**, intermediate **(e)**, and non-classical (**f**) monocytes expressing ICAM-1/CD54 in healthy subjects, patients with end-stage renal disease (ESRD), hemodialysis (HD), peritoneal dialysis (PD), and kidney transplantation (KT). MFI: mean fluorescence intensity. Statistical significance was denoted by *p≤0.05, **p≤0.01, ***p≤0.001 vs HS; $^{\#}$p≤0.05 vs ESRD.

Supplementary Table

Table S1. Effect of frailty on the immune cells in end-stage renal disease, hemodialysis, peritoneal dialysis, and kidney transplantation patients.

	Fit	Vulnerable	Frail
<u>End-stage renal disease</u>			
**T-helper cells (CD4, %)**	49±10	46±10	43±8 a^T
**T-cytotoxic cells (CD8, %)**	23±9	24±8	31±10 a^T
**CD4/CD8 ratio**	2.5±1.2	2.2±1.2	1.6±0.7 a
<u>Hemodialysis</u>			
**Natural killer cells (cells/μL)**	234±99	105±56 a	185±70 b^T
**Intermediate monocytes (%)**	13±6	20±1 a^T	10±4 b
**CD54+ classical monocytes (%)**	87±11	91±1	77±77 aa
**CD54+ non-classical monocytes (%)**	91±8	65±30	81±13 a^T
**CD54+ classical monocytes MFI**	210±82	183±89	145±56 a^T
**CD54+ non-classical monocytes (MFI)**	199±72	152±86	129±61 a
<u>Peritoneal dialysis</u>			
**T cells (cells/μL)**	1163±438	956±365	786±447 a
**CD54+ classical monocytes (%)**	91±9	95±7	84±8 a^T, bb
**CD54+ intermediate monocytes (%)**	98±4	99±1	96±5 bb
**CD86+ classical monocytes (%)**	87±9	92±10 a^T	86±7 b^T
**CD86+ non-classical monocytes (%)**	71±23	85±20 a^T	74±28
**CD86+ non-classical monocytes (MFI)**	184±34	167±89	209±46 b
<u>Kidney transplant patients</u>			
**T cells (cells/μL)**	1147±625	1045±327	1607±592 a^T, b^T
**T-cytotoxic cells (cells/μL)**	531±313	380±173	841±409 a^T, bb
**B cells (cells/μL)**	147±104	92±43 a^T	127±85
**Natural killer cells (cells/μL)**	187±152	296±184 a^T	200±134
**CD54+ classical monocytes (%)**	89±9	95±8 a^T	94±4
**CD86+ intermediate monocytes (%)**	95±8	98±3 a	91±13

Alternative nomenclature: CD54/ICAM-1, CD86/B7.2.

Statistical significance was denoted by a^T 0.9≥ p >0.05, a p≤0.05, aa p≤0.01, aaa p≤0.001 vs fit; b^T 0.9≥ p >0.05, b p≤0.05, bb p≤0.01, bbb p≤0.001 vs vulnerable.
